# Mediators between Positive and Negative Parenting and Child Depressive and Anxious Symptoms: Findings from a Diverse, At-Risk Sample

**DOI:** 10.3390/children9030350

**Published:** 2022-03-03

**Authors:** Christina M. Rodriguez, Anjali Gowda Ferguson, Samantha Gonzalez

**Affiliations:** 1 Department of Psychology, University of Alabama at Birmingham, Birmingham, AL 35294, USA; sgonzo16@uab.edu; 2 Department of Psychiatry, Virginia Commonwealth University, Richmond, VA 23284, USA; asg5071@gmail.com

**Keywords:** internalizing symptoms, childhood depression, childhood anxiety, harsh parenting, negative parenting, positive parenting, self-concept, attributional style

## Abstract

Background: Although children’s depressive and anxious symptoms have been broadly construed as internalizing problems, the current study sought to identify factors that may differentially contribute to these two mental health problems in a high-risk sample. Prior research has not adequately tested both depressive versus anxious symptoms simultaneously, nor has it adequately considered the role of negative versus positive parenting simultaneously, thereby neglecting the potential overlap in both sets of constructs. Overlooking such potential statistical overlap obfuscates how factors may differentially contribute to either depressive versus anxious symptoms. Existing research has also focused on lower-risk community samples. Method: The present study investigated whether children’s negative self-concept or maladaptive attributional style mediated the link between both negative and positive parenting in a racially diverse, at-risk sample of 65 primary school-age children recruited from mental health agencies. Results: When tested together, more negative parenting, but not less positive parenting, retained direct effects on both depressive and anxious symptoms. Both negative self-concept and maladaptive attributional style fully mediated the association between less positive parenting and children’s depressive symptoms, whereas positive self-concept, but not attributional style, mediated between less positive parenting and anxious symptoms. Conclusions: The current findings underscore potential differential intervention targets for these two internalizing problems and highlight the need for future research to consider both depressive and anxious symptoms, and related predictors, simultaneously to control for their shared variance.

## 1. Introduction

A substantive minority of primary school children experience internalizing problems, with rates at 22% of children self-reporting these problems across several European countries [1] and 8% of children diagnosed with an anxiety or depressive disorder in the U.S. nationally [2]. Depression and anxiety in children are typically construed as expressions of the broadband construct of internalizing behavior problems, potentially because of shared communalities [3,4] or because of reciprocal influences on each other across time [5]. Nonetheless, theoretically, depression and anxiety may entail distinct processes, consistent with the classic cognitive content specificity hypothesis [6]. Despite commonalities, several have argued that childhood depression and anxiety may follow different pathways [7], with particular attention on potential divergences in cognitive processes [8,9,10]. Delineating differential processes can distinguish the contributors to children’s depression versus anxiety.

Relevant cognitive processes include children’s negative self-concept and maladaptive attributional style. Attributional style theory contends that ascribing negative events to internal, stable, and global reasons is maladaptive, contributing to children’s psychopathology [11]. Indeed, exhibiting such a negative attributional style is associated with children’s depressive symptoms [12,13]. Some have extended these findings to anxiety, observing that children’s anxiety symptoms are also linked to a maladaptive attributional style [14]. However, the maladaptive attributional style pattern may be more characteristic of depression rather than anxiety [15]. For example, negative attributional style is more strongly implicated in depressive rather than anxious symptoms in primary school-age and pre-adolescent children [8,10], with others finding no association between anxiety and maladaptive attributional style [16]. Negative attributional style was no longer associated with children’s anxious symptoms when controlling for depressive symptoms [17], suggesting that any observed associations of attributional style with anxiety may reflect the symptom overlap between depression and anxiety. The evidence therefore suggests that negative attributional style may be distinctive to depression rather than anxiety.

In contrast, self-concept appears strongly associated with both depressive and anxious symptoms. Depressive symptoms are significantly related to negative self-concept in 7–10-year-old children [18], with meta-analytic evidence that low self-concept is linked to the development of both depression and anxiety across the lifespan [19]. Depression, anxiety, and negative self-concept were interrelated in pre-adolescents [20] and primary school-age children [8].

Therefore, although negative attributional style may be unique to depressive symptoms, negative self-concept may represent a commonality between children’s depression and anxiety. Maladaptive attributional style patterns and negative self-concept are modestly interrelated in primary school-age samples of children [21,22]. Empirically examining depressive and anxious symptoms concurrently may clarify the roles of attributional style and negative self-concept in either depressive or anxious symptoms (or both) as two separate cognitive contributors to the development of these internalizing problems.

Other research on the development of children’s internalizing symptoms has implicated parenting processes. Whereas positive parenting encompasses warmth, affection, sensitivity, and responsiveness [23], negative parenting is viewed as harsh, authoritarian, rejecting, controlling, or abusive [24]. Although some presume that positive and negative parenting are polar opposites, the evidence recognizes that positive and negative parenting are related but orthogonal, distinct modalities [25,26,27]; in other words, parents may engage in both negative and positive parenting behaviors with their children.

When examined simultaneously, both low positive parenting and high negative parenting were related to children’s depressive symptomatology in a sample of children ages 7–11 [25]. Mothers’ reports of less positive parenting behavior were linked to children’s self-reported depressive symptoms but not to mothers’ reports of negative parenting, although this study utilized separate statistical models for the parenting behaviors and thus did not test the differential effects of parenting behavior [27]. Turning to research that has included children’s anxious symptoms in relation to parenting, one meta-analysis reviewing studies of 5–12-year-old children indicated that less warmth and more harsh, abusive parenting was associated with depressive symptoms and internalizing symptoms in general, with more equivocal effects observed for children’s anxious symptoms [24]. When tested simultaneously, depressive and anxious symptoms were related to different negative perceptions of parental discipline in 7–11-year-old children [8]. Another community sample of children considered both anxious and depressive symptoms simultaneously, identifying that children’s perceptions of maternal rejection and control were related to both anxiety and depression, but more strongly to depressive symptoms when controlling for anxiety [28]. Collectively, these findings suggest that there may be differential effects of positive and negative parenting on children’s depressive versus anxious symptoms when tested simultaneously, potentially with more consistent effects in relation with children’s depressive symptoms.

Although the literature is limited in testing how cognitive factors may account for the link between parenting and internalizing problems, harsher parental discipline and greater child abuse risk are positively related to primary school-age children’s negative attributional style, negative self-concept, depression, and anxiety [29,30]. Research suggests that children’s negative attributional style mediates the association between parent-reported greater harsh parenting and child abuse risk with children’s reported internalizing symptoms [31]. Negative attributional style also may mediate the association between greater maternal parenting control and children’s anxiety symptoms [14]. Another study observed that negative parenting was associated with poor self-concept, which, in turn, linked to symptoms of anxiety and depression, but positive parenting linked primarily to better self-concept [32]. Thus, the extant research ties negative parenting to both poor self-concept and maladaptive attributional style, and to both depression and anxiety, with limited research and less clarity on how positive parenting relates to either attributional style or self-concept for both depression and anxiety.

Yet, most of the aforementioned research has involved community samples of parents and children, which may obscure how harsh or positive parenting and cognitive processes contribute to clinical levels of children’s internalizing symptoms. Parents who experience mental health problems are recognized to be at considerable risk for engaging in negative parenting [33,34,35]. Furthermore, children with mental health problems are more likely to experience harsh parenting [36,37,38].

**Figure 1:**
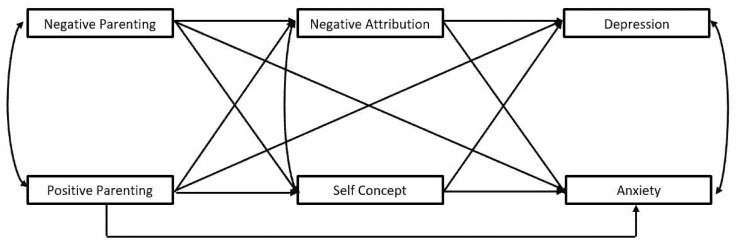
Proposed path model.

Consequently, the current study focused on an at-risk sample of children who were receiving mental health treatment or whose mothers were receiving mental health treatment. Thus, this study focused on a clinical sample of children wherein the potential effects of interest regarding the links with negative or positive parenting would be magnified and theoretically most relevant for those working with children and families in clinical settings. The primary goal was to examine whether children’s reports of negative versus positive parenting related to depressive versus anxious symptoms mediated by negative self-concept and maladaptive attributional style. To parse differential effects, we tested a hypothesized model wherein depressive and anxious symptoms, as well as positive and negative parenting, were tested simultaneously, with the two proposed cognitive processes (self-concept and attributional style) as potential partial mediators (see Figure 1). The study sought to clarify the role of both parenting and cognitive processes that may thereby differentiate the development of children’s depression versus anxiety.

## 2. Materials and Methods

### 2.1. Participants and Procedures

The current study involved a sample of 65 children aged 7–11 recruited from flyers distributed at community outpatient mental health agencies in the Southeast U.S. To be eligible, either the child, mother, or both were required to be receiving treatment for a mental health diagnosis (excluding children whose mothers reported developmental disabilities that could compromise their comprehension of the study measures). Regarding receipt of mental health services, 51.7% of the sample involved children alone receiving treatment, 25.9% involved both the mother and child receiving treatment, and 22.4% involved mothers alone receiving treatment. Interested mothers contacted the lab for eligibility screening and scheduled a 90 min session. All measures were delivered in randomized order and orally administered to children by a trained research assistant in a private area wherein children wrote their responses on their own sheet under supervision of the research assistant. The university Institutional Review Board granted approval for the study and both mothers and children separately provided informed consent.

This sample was comprised of 59.4% males (*M_age_* = 8.63 years, *SD_age_* = 1.74). Mothers reported racial and ethnic composition as follows: 58.9% Black/African American, 33.9% White, and 1.8% Native American, with the remainder identifying as Other; of these, 7.3% also identified as Hispanic/Latina. The median annual household income was between USD 13,000 and USD 19,999, with 59.6% of mothers indicating that they were raising their children as single parents.

### 2.2. Measures

#### 2.2.1. Independent Variables

The Parent Perception Inventory (PPI) [39] is an 18-item self-report measure of children’s experience of positive and negative parenting behaviors. Half of the items involve descriptions of positive parenting behaviors (e.g., positive reinforcement, nurturance, and affection; e.g., “Hug you, kiss you, tickle you, smile at you”), which contribute to a Positive Parenting Scale. The remaining items comprise negative parenting behaviors (e.g., harsh parenting and disapproval; e.g., “Tell you you’re no good; tell you that you messed up or didn’t do something right. Criticize you”), which contribute to the Negative Parenting Scale. Children indicated the perceived frequency of such behaviors from their mothers on a 5-point scale from 0 (never) to 4 (a lot), with a visual thermometer depicting these frequency gradations to facilitate comprehension. Subscale scores were generated by summing across their nine items. Higher Negative Parenting Subscale scores indicate that children perceive their mother as using negative parenting techniques more frequently; higher Positive Parenting Subscale scores indicate that their mother used more frequent positive parenting behaviors. The current sample’s scores demonstrated acceptable internal consistency for both Positive and Negative Parenting Subscales (α = 0.79 and α = 0.69, respectively), comparable to that reported by the test authors. Higher PPI scores are related to parents’ report of harsher parenting [40], demonstrating validity.

#### 2.2.2. Proposed Mediators

The Children’s Attributional Style Questionnaire-Revised (CASQ-R) [41,42] is the most frequently used self-report measure designed to assess children’s attributional style. This version includes 24 items, with half of the items describing positive events and the other half describing negative events. Each item depicts these hypothetical events and the child is asked to select from two reasons why the event occurred. The reasons emphasize one of three dimensions (internality, stability, and globality), holding the other two dimensions constant. The current study focused on children’s responses to the 12 negative events that convey a maladaptive attributional style that ascribes these negative events to internal, stable, and global reasons. Higher CASQ Negative Total scores correspond to a more maladaptive attributional style. Previous research reports moderate test–retest reliability [42] and strong predictive validity for depression [25,43,44], with modest internal consistency (α = 0.50–0.63) [45,46,47,48], similar to that obtained in the current study (α = 0.53).

The Self-Perception Profile for Children (SPPC) [49] is an 18-item measure of children’s self-concept in domains such as personality, behavioral control, and physical appearance. For each item, a two-step process yields a 4-point rating scale; children are first posed two statements (e.g., “Some kids like the kind of person they are” versus “Other kids often wish they were someone else”) and asked to choose which best characterizes them, followed by asking whether their choice is “really true” or “sort of true” of them. Summed across the 18 items, higher scores indicate a more positive self-concept. The SPCC demonstrates good reliability and validity [50]. Scores in the current sample evidenced acceptable reliability (α = 0.85).

#### 2.2.3. Dependent Variables

The Children’s Depression Inventory (CDI) [51] is one of the most frequently used self-report measures of childhood depressive symptoms. The 27 items present three statements reflecting depressive symptoms of varying severity, valued from 0 to 2. Summed across items, higher CDI Total scores convey endorsement of more severe depressive symptoms. Prior research indicates good reliability and diagnostic validity [52], with scores in the current sample demonstrating acceptable reliability (α = 0.85).

The State-Trait Anxiety Inventory for Children (STAIC) [53] is a child-report measure of anxiety. The current study focused on the 20 items involving trait anxiety symptoms. Each item was rated on a 3-point scale, including 1 (hardly ever), 2 (sometimes), and 3 (often), with higher scores suggesting greater dispositional anxiety. Internal consistency and test–retest reliability of the measure is acceptable [53], with evidence of diagnostic validity [54]. Total STAIC scores in the current study demonstrated adequate reliability (α = 0.73).

### 2.3. Analytic Plan

Preliminary statistics were performed with SPSS 27, providing descriptive and correlational findings. To examine the proposed mediation model (Figure 1), Mplus 8.1 was utilized to obtain maximum likelihood estimates of model coefficients using bootstrapping (1000 samples) to provide bias-corrected 95% confidence intervals for direct and indirect effect estimates (confidence intervals excluding zero further affirm a significant coefficient). Ideally, models should demonstrate good fit on multiple indices. Model fit was evaluated using the comparative fit index (CFI), root mean square error of approximation (RMSEA), and the standardized root mean square residual (SRMR) [55]. CFI values above 0.95 and SRMR and RMSEA values below 0.08 suggest good model fit [55].

## 3. Results

### 3.1. Preliminary Analyses

Means and standard deviations for all measures appear in Table 1. In the current at-risk sample, 24.3% obtained clinically elevated CDI total scores above the recommended cut-off of 19 [51] and the mean STAI-C Total score was comparable to those obtained by children with anxiety disorders (cf. [54]). No significant differences by child gender were observed across all measures (given the prepubertal age range of this sample); furthermore, neither household income nor mothers’ educational level (indicators of family socioeconomic status) were significantly associated with any of the measures. Consequently, the remaining analyses proceeded without covariates.

Correlations across measures also appear in Table 1. These preliminary results affirm a strong positive association between children’s self-reported depressive and anxious symptoms, and a modest association between children’s perception of less positive parenting and high negative parenting behavior. More positive parenting behavior was associated with better self-concept and a lower likelihood of a negative attributional style, whereas perceived negative parenting behavior was not significantly associated with self-concept or attributional style.

### 3.2. Path Model Results

**Figure 2:**
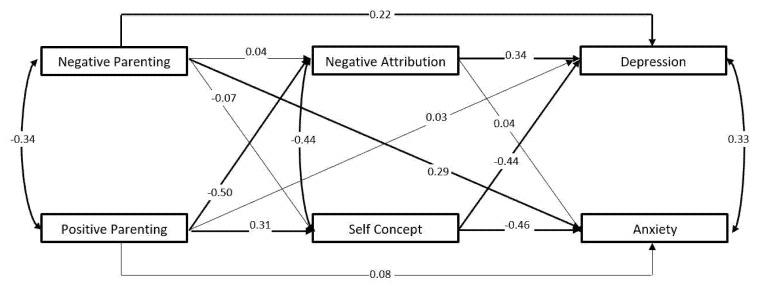
Standardized path coefficients.

The model depicted in Figure 1, with depressive and anxious symptoms serving as dependent variables simultaneously, demonstrated an adequate model fit: CFI = 0.99, SRMR = 0.03, RMSEA = 0.10 (see Table 2 for standardized path coefficients with confidence intervals in brackets and Figure 2 for standardized path coefficients where bold lines indicate statistically significant paths). Whereas negative self-concept was associated with both depressive and anxious symptoms, a maladaptive negative attributional style was significantly linked to depressive but not anxious symptoms. Better self-concept and less maladaptive attributional style were directly linked to more perceived positive parenting (but not associated with less negative parenting). Only perceived negative parenting, not positive parenting, retained significant direct links to both depressive and anxious symptoms. Observed indirect effects (see bottom of Table 2) indicated that depressive symptoms were indirectly related to positive parenting through maladaptive negative attributional style and poorer self-concept (fully mediated). For anxious symptoms, although not directly related to less positive parenting, children’s negative self-concept also demonstrated an indirect effect. (Given the cross-sectional nature of the design, we probed a reversed path model wherein children’s depressive and anxious symptoms would predict positive or negative parenting as dependent variables. However, that path model demonstrated that neither depressive or anxious symptoms were significantly linked to either positive or negative parenting, with only less maladaptive attributional style remaining as significantly associated with more positive parenting. Full results are available upon request.)

## 4. Discussion

The present investigation considered whether negative or positive parenting (tested simultaneously) would demonstrate differential effects on depressive or anxious symptoms through self-concept and attributional style with an at-risk, diverse sample of children. The current findings suggest that children’s perceptions of negative parenting retained direct effects on their depressive and anxious symptoms, whereas positive parenting did not when controlling for negative parenting. In contrast, children’s negative self-concept and maladaptive attributional style mediated the association between less positive parenting and children’s depressive symptoms, whereas positive self-concept mediated the relation between positive parenting and children’s anxious symptoms.

Using a well-controlled statistical model, the current findings indicate that negative parenting directly relates to symptoms of both depression and anxiety, controlling for positive parenting. These findings are generally consistent with prior research with community samples on the role of negative parenting in relation to depressive symptoms and anxiety (e.g., [25,29]). Our findings also align with those demonstrating that positive and negative parenting are related but distinct constructs [25,27]. In contrast to our findings, others had suggested that less mother-reported positive parenting, not more negative parenting, was associated with depressive symptoms in separate statistical models [27]. These studies differ from the current study in several notable ways given that the vast majority of the literature relies on non-clinical samples and typically utilizes statistical models that do not control for potential overlaps between either positive and negative parenting or between anxiety and depressive symptoms. For example, the current study observed that less positive parenting was significantly associated with children’s depressive symptoms in simple bivariate analyses but the model controlling for negative parenting indicated that positive parenting did not exert direct effects, only indirect effects. Furthermore, the current study utilized children’s perceptions of their parenting (cf. [27]); although a multi-informant design has advantages, arguably a child’s internalizing symptoms may be more likely influenced by their *perceptions* of parenting rather than parents’ potentially biased reports of their parenting behavior. Furthermore, note that negative parenting did not exert indirect effects through either negative self-concept or attributional style, suggesting that other unstudied mechanisms may need to be identified in future work to understand exactly how negative parenting contributes to internalizing problems. In sum, our findings underscore the relatively more direct role of perceived negative parenting in the development of internalizing symptoms. Consequently, programs aimed at reducing harsh parenting techniques could diminish the likelihood of children developing internalizing symptoms.

Findings that less perceived positive parenting was indirectly associated with greater depressive and anxious symptoms through negative self-concept affirm prior work highlighting the potential role of self-concept as a commonality in both depression and anxiety [8,20]. Promotion of positive parenting could provide substantial value to children potentially beyond protective effects on their mental health given that positive self-concept is likely to result in a host of benefits for children’s overall functioning. This study also supports the growing evidence that negative attributional style is more specific to the development of depressive and not anxious symptoms [10,15,16], representing a factor distinguishing these two related but distinct childhood mental health problems. In the present investigation, negative attributional style indirectly accounted for the relation between perceived low positive parenting and children’s depressive symptoms. This supports the contention that children’s explanatory style for negative events represents a relevant focus within interventions for child depression, in particular, rather than for anxiety. Together, the findings on these two cognitive factors highlight the continued need for additional research that identifies alternative mechanisms that may link parenting to the differential development of children’s depression versus anxiety.

The current study’s findings are preliminary given its small sample size. Furthermore, the findings derive from a cross-sectional study of data obtained from a single source about mothers’ parenting only. A critical feature to include in future research is the role of fathers’ negative and positive parenting, again tested simultaneously to disentangle the father’s contributions to children’s depression versus anxiety. Although children’s internalizing problems may arise from their perception of the parents’ behavior (the competing possibility that internalizing problems contribute to perceived parenting was inferior), directional effects cannot be established. However, the current study’s preliminary findings point to a number of interesting future research directions. By utilizing a larger clinical sample, future studies could consider a multi-informant approach, gathering the perspective of children and parents (both mothers and fathers, where fathers are present) about parenting behavior as well as about internalizing symptoms to empirically compare findings based on the reporting source. Current research highlights that parents’ report of behavior problems diverge considerably from their children’s reports, particularly regarding internalizing problems (e.g., [56]). Such a study would also ideally entail a longitudinal design to clarify whether parenting behavior precedes a proposed larger collection of potential mediators that, in turn, precede the development of depressive versus anxious symptoms, again employing a well-controlled statistical model. Such larger studies should consider incorporating broader assessments of parenting practices, including the role of perceived parent–child attachment. Such future work would provide insight into whether the current preliminary findings with this at-risk sample are replicated.

## 5. Conclusions

Given the scope of children’s internalizing problems in the U.S. [2], the current findings point to potential differential processes in depression versus anxiety, supporting prior arguments of these as separable disorders [8,9,10]. This raises questions about collapsing into a single “internalizing” domain, which could hamper efforts to pinpoint the processes that may differ between them. For example, although reducing negative parenting and promoting positive parenting as well as children’s positive self-concept remain important targets for intervention for both depression and anxiety, addressing maladaptive attributional style would be more suitably targeted for depression. Moreover, the current findings highlight that negative and positive parenting are also distinct; some programs focus predominantly on reducing negative parenting while neglecting strategies for how to encourage positive parenting. The role of both parenting dimensions appears to be important to address in family interventions. The present investigation focused on the potential role of perceived parenting and two cognitive factors, but additional processes need to be identified to better respond to the needs of children dealing with these mental health problems. As we continue to detect processes that distinguish depression and anxiety, interventions can thereby become more tailored.

## Figures and Tables

**Table 1:** Means, standard deviations, and intercorrelations across measures.

		*M* (*SD*)	2.	3.	4.	5.	6.
1	PPI Positive Parenting	26.53 (6.62)	−0.34 **	−0.52 ***	0.33 **	−0.42 ***	−0.19
2	PPI Negative Parenting	16.13 (6.70)		0.21	−0.18	0.37 **	0.35 **
3	CASQ Negative	2.86 (2.01)			−0.30 *	0.52 ***	0.20
4	SPPC Self-Concept	56.10 (9.75)				−0.58 ***	−0.49 ***
5	CDI Depression	13.62 (7.94)					0.55 ***
6	STAIC Trait Anxiety	39.06 (7.31)					

Note. PPI Positive = Parent Perception Inventory, Positive parenting subscale; PPI Negative = Parent Perception Inventory, Negative parenting subscale; CASQ = Children’s Attributional Style Questionnaire; SPPC = Self-Perception Profile for Children; CDI = Children’s Depression Inventory; STAIC = State-Trait Anxiety Inventory for Children. * *p* ≤ 0.05, ** *p* ≤ 0.01, *** *p* ≤ 0.001.

**Table 2:** Standardized direct and indirect path coefficients with confidence intervals.

	*β* (CI)	*p*
**Direct effects**		
PPI Pos Parenting → CDI Depression	0.03 [−0.23, 0.16]	0.776
PPI Neg Parenting → CDI Depression	**0.22 [0.04, 0.39]**	**0.039**
SPPC Self-concept → CDI Depression	**−0.44 [−0.25, −0.63]**	**0.000**
CASQ Negative → CDI Depression	**0.34 [0.15, 0.53]**	**0.004**
PPI Pos Parenting → STAIC Anxiety	0.08 [−0.15, 0.31]	0.575
PPI Neg Parenting → STAIC Anxiety	**0.29 [0.07, 0.51]**	**0.032**
SPPC Self-concept → STAIC Anxiety	**−0.46 [−0.26, −0.66]**	**0.000**
CASQ Negative → STAIC Anxiety	0.04 [−0.11, 0.18]	0.679
PPI Pos Parenting → CASQ Negative	**−0.50 [−0.35, −0.66]**	**0.000**
PPI Neg Parenting → CASQ Negative	0.04 [−0.13, 0.21]	0.704
PPI Pos Parenting → SPPC Self-concept	**0.31 [0.10, 0.51]**	**0.014**
PPI Neg Parenting → SPPC Self-concept	−0.07 [0.12, −0.27]	0.538
STAIC Anxiety ↔ CDI CDI Depression	**0.33 [0.11, 0.56]**	**0.016**
PPI Neg Parenting ↔ PPI Pos Parenting	**−0.34 [−0.14, 0.54]**	**0.006**
**Indirect effects**		
PPI Pos Parenting → CASQ Negative → CDI Depression	**−0.17 [−0.07, −0.28]**	**0.007**
PPI Pos Parenting → SPPC Self-concept → CDI Depression	**−0.13 [−0.04, −0.23]**	**0.017**
PPI Neg Parenting → CASQ Negative → CDI Depression	0.01 [−0.05, 0.08]	0.731
PPI Neg Parenting → SPPC Self-concept → CDI Depression	0.03 [−0.05, 0.12]	0.530
PPI Pos Parenting → CASQ Negative → STAIC Anxiety	−0.02 [−0.10, 0.06]	0.694
PPI Pos Parenting → SPPC Self-concept → STAIC Anxiety	**−0.14 [−0.03, −0.25]**	**0.029**
PPI Neg Parenting → CASQ Negative → STAIC Anxiety	0.00 [−0.02, 0.02]	0.896
PPI Neg Parenting → SPPC Self-concept → STAIC Anxiety	0.03 [−0.06, 0.12]	0.537

Note. PPI Pos = Parent Perception Inventory, Positive Parenting subscale; PPI Neg = Parent Perception Inventory, Negative Parenting subscale; CASQ = Children’s Attributional Style Questionnaire, Negative; SPPC = Self-Perception Profile for Children; CDI = Children’s Depression Inventory; STAIC = State-Trait Anxiety Inventory for Children (Trait). **Bolded** values are statistically significant.

## Data Availability

The data presented in this study are available upon request from the corresponding author. The data are not publicly available in accordance with the consent provided by participants and as approved by the Institutional Review Board.
